# Global justice in the context of transnational surrogacy: an African bioethical perspective

**DOI:** 10.1007/s11017-022-09581-4

**Published:** 2022-07-25

**Authors:** Ademola Kazeem Fayemi, Amara Esther Chimakonam

**Affiliations:** 1 Australian Catholic University, Brisbane, Australia; 2 University of Lagos, Akoka, Nigeria; 3 University of Johannesburg, Johannesburg, South Africa

**Keywords:** Global justice, Transnational commercial surrogacy, Afro-communal justice, Global South

## Abstract

The ongoing debate on how best to regulate international commercial surrogacy defies consensus, as the most cogent normative and jurisprudential grounds for and against non-altruistic surrogacy remain controversial. This paper contributes to the debate by focusing on social justice issues arising from transnational, moneymaking surrogacy, with a focus on the Global South. It argues that existing theoretical perspectives on balancing interests, rights, privileges, and resources in the context of cross-border surrogacy—such as cosmopolitanism, communitarianism, liberal feminism, radical feminism, and neorealism—are not sufficient to treat the question of justice underpinning transnational surrogacy in the Global South. An Afro-communal theory of social justice is proposed as an alternative model for addressing the shortcomings in existing global justice theories. Building on Thaddeus Metz’s construction of Afro-communal social theory and a bioethic of communion, this article reveals the fundamental nature of injustices in the Global South surrogacy foray. This approach provides prima facie grounds for making commercial surrogacy more just in the evolving global order.

## Introduction

This paper explores the question of global justice in the context of transnational surrogacy from an African bioethical perspective. Transnational surrogacy, in the form of commercial cross-border assisted reproductive services by women from the Global South, is a widespread practice with implications that warrant ethical reflection [1–4]. The massive drift toward the Global South in efforts to recruit surrogate agents is, among other things, a function of unbalanced global structures of resource distribution, social exclusion, and economic inequalities that are rife in the contemporary global order. Economic globalization has helped to sustain global inequality. Just as the Global South has constituted a marketplace for raw materials sourced for finished products in the Global North, so has it also served as a cheap market for transnational surrogacy.^1^

Given the upsurge in Global North transnational surrogacy companies foraying into the Global South for profit maximization, the question of justice lurks at the center of non-altruistic (or paid) international surrogacy encounters. Expressed in simple terms, the question is: what is the most cogent normative position that addresses issues of justice in the practice of non-altruistic surrogacy in transcontinental and globalized contexts? The globalization of reproductive rights poses a unique problem for global justice—if autonomy, freedom, and equality are the notions by which the international community understands citizenship rights and responsibilities, what are the implications for transnational commercial surrogacy in the Global South in view of existing structural inequalities between South and North? Many political philosophers—from cosmopolitans to communitarians, feminists to neorealists—have attempted to answer these questions.^2^

In this paper, we offer a fresh perspective on these issues, arguing that existing theoretical approaches to balancing interests, rights, privileges, and resources in the context of cross-border surrogacy are not sufficient to address the question of justice underpinning transnational surrogacy practices in the Global South. Instead, we develop an Afro-communal theory of social justice as an alternative model for addressing the shortcomings of philosophical theories of global justice. This Afro-communal approach is integral to Thaddeus Metz’s relational ethic [5], which provides prima facie grounds for making transnational commercial surrogacy more just in the evolving global order.

The paper is structured in four parts. The first part is a conceptual exercise on the meaning of transnational surrogacy. The second part discusses the nexus between global inequality and transnational surrogacy in relation to the Global South. The third part critically surveys cosmopolitan, communitarian, liberal feminist, radical feminist, and neorealist perspectives in the debate on justice and transnational surrogacy. The final part presents an Afro-communal theory of social justice that addresses the weaknesses of the surveyed theories. In particular, we will deploy Metz’s interpretation of justice within an Afro-communal theory as a way of remedying the injustices of commercial surrogacy in the global system.

## Surrogacy and its types

Surrogacy is one major type of assisted reproductive technology, which is aimed at overcoming infertility and realizing one’s desire to have children by means other than adoption. It refers to a situation in which a third-party female agrees to become pregnant, carry a fetus, and give birth to a child on behalf of another person or persons, over to whom she transfers the child at birth. In other words, surrogacy is a process in which an intended parent or couple enters into a reproductive agreement with a third party predicated on the latter’s handing over the child after birth and relinquishing all parental rights thereto. Opting for surrogacy seems to have some relative advantages over adopting for prospective parents: “While adoption may satisfy one’s desire to provide nurturance for a child, adoption cannot satisfy the yearning to create the child and to watch as a version of oneself unfolds and develops” [6, p. 389]. Surrogacy, unlike adoption, enables one to separate rearing from pregnancy and childbearing without forgoing the goods of biological connection. The desire to have genetic offspring in spite of reproductive challenges is a driving force behind surrogacy.

Some surrogacy arrangements involve in vitro fertilization (IVF) through injection of semen into the surrogate’s body by means other than sexual intercourse; other arrangements involve transferring a fertilized embryo directly into the surrogate’s uterus. The former type of arrangement is known as traditional surrogacy, while the latter is known as gestational surrogacy. In traditional surrogacy, the surrogate contributes her own egg, and the sperm of a commissioning father is inserted into the surrogate’s vagina through a method known as artificial or intrauterine insemination. Thus, both the surrogate and the commissioning father have genetic links to the child. This genetic bond often results in the surrogate’s having feelings of attachment to the child, which can make her reluctant to relinquish the child after birth and thereby lead her to breach the surrogacy agreement. Not only do such cases give rise to legal tussles such as the famous *Baby M* case [7],^3^ but they also raise moral issues concerning parental rights, children’s well-being, birthing mothers’ right to privacy and autonomy, and the commodification of human biological functions [8, 9].

By contrast, in gestational surrogacy there is no genetic connection between surrogate and fetus. Rather, the surrogate’s uterus is implanted with an embryo that is created with the egg and sperm of either commissioning parents or donors using a method known as in vitro fertilization (IVF). With this method, doctors usually administer drugs to a commissioning parent or donor in order to stimulate the development of multiple ovarian follicles, which are collected and fertilized in a laboratory using sperm from another commissioning parent or donor, and the fertilized embryo is then transferred into the surrogate’s uterus. In this case, the surrogate is not biologically related to the child; she only gestates the fetus to term.

The preference for either traditional or gestational surrogacy is a function of the perceived overriding importance of the affordances of one versus the other—a perception that differs considerably across cultures and gender divides. Infertile couples might opt for gestational surrogacy as it makes one or both of them genetically linked to the child and provides them more legal rights since the surrogate is not in any way genetically bond to the child. At the level of gender, more women tend to prefer gestation to genetics, while more men favor genetic tie to the gestational tie [10].

A great challenge to gestational surrogacy is the unpredictability of the attitudes of the intended parents and surrogates toward pregnancy complications and outcomes. In cases where gestational surrogacy results in abnormal complications that threaten the surrogate’s life or in multiple births, as is often with artificial reproductive technology pregnancies, or in physically challenged babies, the attitude of both parties may differ. For instance, in the case of a physically challenged baby, intended parents may want to revoke the contract against the wish of the surrogate which might leave the surrogate with the financial burden and moral responsibility of looking after a physically challenged child. The surrogate might want to relinquish the child to the intended parents as an end to the pregnancy contract. This raises a major concern about the responsibility to a physically challenged child and the rights of such a child within a surrogacy contract: who should be socially and morally responsible for children with physical challenges within surrogacy arrangements?

Surrogacy services can be offered either on altruistic or on commercial grounds. On the one hand, in altruistic service, the surrogate offers to help the intended couple out of compassion, empathy, and sympathy without collecting any financial compensation in return. This form of service is prevalent in traditional surrogacy, where most of the contracts are entered on an altruistic level. On the other hand, commercial service involves a surrogate being paid a fee for the surrogacy service she renders. Today, commercial surrogacy is more popular than altruistic surrogacy. This seems to be the case because of the high rate of infertility among couples; other relevant factors include health issues which might impair childbearing, homosexuality, the rise in artificial reproductive technologies (ARTs), legal parental right, genetic ties.

Surrogacy is faring well in the Global South because of the relatively low cost of obtaining surrogacy services compared to in Global North, specifically the United States. For example, “[a] surrogacy service in India costs about $11,000 including in vitro fertilization (IVF) charges while in the USA, surrogacy alone, excluding ART charges costs about $15,000, approximately” [11, p. 141]. Similarly, the monetary incentives that accompany surrogacy coupled with the economic hardship among women in the Global South continue to drive the surrogacy industry. Compared to surrogates in the United States, who earn about $25,000–$40,000 with other incentives such as “a post-birth grace period, a pre-birth opt out clause, compensation for expenses, quality medical care, legal representation, and the opportunity for an ongoing relationship with the child and its adoptive family” [3, p. 332], the estimated compensation for Indian women is around $3000–$6000 [12, p. 690]. This monetary incentive for Indian women can be used to offset debt, feed the family, and attain a level of social well-being. Scholars have used different words to capture this phenomenon, such as “global reproductive market” [13], “cross border reproductive care” [12], and “transnational surrogacy” [14]. However, all these phrases about surrogacy involve outsourcing for gestation, egg donors, and surrogates. Our paper uses transnational surrogacy to mean the globalization of reproductive care. Transnational surrogacy “is an intimate industry that entails a bureaucratized movement of hundreds of thousands of individuals who crisscross the globe in pursuit of fertility assistance, human eggs, and sperm” [14, p. 941].

## Global inequality, transnational surrogacy, and the Global South

Disparities in income and asymmetrical distribution of wealth among states and citizens in different parts of the world have been at the forefront of the discourse on global inequality [15, p. 1]. Using the 2016 International Monetary Fund data for per capita gross domestic product GDP, adjusted for purchasing power parity (PPP), which is considered a better way of comparing the living standards and poverty lines among the world’s richest and poorest countries, the richest 10% of countries in the world have an average GDP-PPP that is 74 times higher than the poorest 10% of countries, many of which are found in sub-Saharan Africa [16]. In other words, there are wide disparities in the living conditions between the Global South (Asia, Africa, and Latin America) and other parts of the world.^4^ These disparities reflect the uneven distribution of global resources that concentrate wealth in the hands of the Global North.

According to Stephen Krasner, in the global sphere, there are “makers, breakers, and takers” [18]^5^; and unfortunately, the Global South plays the takers role whereas the Global North plays the makers and breakers roles. As takers, the Global South enters into unequal economic relationships with the developed world that impair their economic growth and development with the ugly results that they must “accept rules written by—and usually for—the more developed countries” [19, p. 5]. These “rules being written into multilateral and bilateral agreements actively prevent developing countries from pursuing the kinds of industrial and technology policies adopted by the newly developed countries of East Asia, and by the older developed countries when they were developing” [20, p. 622]. This “shrinking of development space” [20] by the Global North creates extreme economic inequality in the Global South, with heavy consequences for the poor.

Sustaining such inequality in a globalized world economy in which the Global North acts as a market for raw materials and finished products poses a challenge [21, 22]. On the one hand, globalization serves to increase the economic interdependence of states across the globe through the increasing volume and size of cross-border transactions in goods and services and flow of international capital, and through the more rapid and widespread diffusion of technology. On the other hand, globalization has brought about a rise in global inequality through a subtle fostering of “financial volatility, job insecurity, health insecurity, environmental issues, and political conflicts” in poor countries [23, p. 20]. Thus, the Global North accrues the goods of globalization while the Global South experiences greater disadvantages with a high rate of poverty.

The effect of this economic stratification on unequal global relations in the context of transnational surrogacy is manifold. First, the Global South serves as a cheap market for transnational surrogacy. There has been an upsurge in the presence of Global North transnational surrogacy businesses into the Global South with the aim of profit accumulation. Here again, the Global South is placed in the position of the takers. Unequal economic relations, and the minimal role played by the Global South in such relations, has pushed most of its populace deeper into poverty. As Ethan Kapstein observes:

Today, when we hear or read about the global economy, it is usually in terms of the trillions of dollars of goods, services, and investment that circle the planet, with the great increases in national wealth that accrue to states that adopt open policies. But there are other data that usually go unnoticed in these discussions. We hear less about growing income inequality. And we hear still less about the 1.3 billion people in the developing world whose income level is under $1 per day. For all these people, the global economy has not yet brought either material gifts or the hope of a better life. [24, p. 16]

Studies have shown that women are the most affected in the global economic order. A sizable population of women in the Global South still play minimal economic roles. For instance, “in Bangladesh, women account for almost 85% of workers in the garment industry” and “the poorest rural women are almost six times more likely … to never attend school” [17, pp. 10–11]. These women in poverty serve as cheap labor for transnational surrogacy in the Global North. Many young women of childbearing age in the Global South engage in commercial surrogacy (whereas, comparably, the Global North has a small number of surrogates) as a means of livelihood. Empirical studies have shown that surrogates in the United States are likely to be college graduates who are not financially motivated but opt to become surrogates because surrogacy affords them the opportunity to give the ultimate gift of life (children) to others and the time to pursue their other interests; by contrast, Indian surrogates are relatively uneducated, often illiterate, and poor individuals, who are motivated to become surrogates because they need the money (see [25–27]). As a result, many surrogates in India seem to have lesser bargaining power during a surrogacy contract, unlike their counterparts in the United States, whose level of education enables a better understanding of and bargaining power during surrogacy arrangement (see [3]).

The extreme structural inequalities seen in the practice of transnational surrogacy, however, are not restricted to surrogacy alone but extend to virtually all global structures. Thus, structural inequality remains one of the motivating factors behind surrogacy today. Many believe that the root cause of this structural inequality is the globalization of reproductive services [28], reinforced by the liberalist framework grounding reproductive rights. Yet there are other ethical frameworks that enjoy less global prominence than liberalist principles that originate from the West. One implication of the privileging of Western frameworks is that the strong appeal to rights in the debate about global justice for transnational surrogacy tends to perpetuate the North–South inequities in the surrogacy industry.

Second, the advent of globalization has resulted in the commercialization of reproductive rights beyond borders, which, in turn, has given rise to childbearing and nurturing cultures around the globe—with the Global South at the frontier. The world has become so interwoven that people can extend their reproductive rights beyond their particular territory or jurisdiction. Currently, there is a massive contingent of intended couples moving from the Global North to the Global South in hopes of fulfilling their childbearing wishes at the expense of the well-being of the impoverished surrogates, who engage in commercial surrogacy as an attempt to quickly escape poverty [22, p. 690]. Although scholars like Joseph Stiglitz, and others within his camp, strongly believe that “globalization does not have to be bad for the environment, increase inequality, weaken cultural diversity, and advance corporate interests at the expense of the well-being of ordinary citizens” [29, p. xv], the reality has shown a transitive relationship between globalization, inequality, and poverty.

The key questions are twofold. Does the globalization of reproductive rights evince a unique problem for global justice? If citizenship, freedom, and equality are the notions by which international society understands citizenship rights and responsibilities, what does this mean for transnational commercial surrogacy in the Global South given the structural inequality that exists between the North and South? Many political philosophers, from cosmopolitans to communitarians, liberal feminists, radical feminists, and neorealists, have attempted to answer these questions.

For cosmopolitans, the elimination of such structural inequalities and redistribution of wealth will guarantee global justice. Liberal feminists want a regime of rights and freedoms, including the right to exercise a reproductive option that diffuses an economic system controlled by the patriarchy to open better opportunities, including surrogacy, for women’s empowerment. Radical feminists conceive of surrogate motherhood as a form of patriarchal denigration and objectification of women in a presently male-dominated world. Communitarians and neorealists, although with differences, insist that a state-centric approach to transnational commercial surrogacy must be adopted in order to achieve global justice. Fundamentally, to what extent do existing theoretical perspectives on global justice provide grounds for a fair and just system of transnational surrogacy?

## Transnational surrogacy and the question of global justice

Issues of justice in transnational surrogacy have to do with balancing the moral entitlements, duties, rights, and interests of the various stakeholders (commissioning couples, physicians, surrogacy agencies) involved in the phenomenon in ways that avoid exploitation of the surrogate mother and respect both the dignity of the child and shared future humanity. The dominant theoretical perspectives in the contentious debate on the question of justice in transnational surrogacy include cosmopolitanism, communitarianism, neorealism, and radical and liberal feminism.

Cosmopolitanism presupposes that individuals are to be world citizens belonging to a global society [30]. Although, there are many variants of cosmopolitanism [31], the focus of this paper is right cosmopolitanism. Operating within the ideals of cosmopolitanism, right cosmopolitanism states that “Parties that impose an institutional scheme that foreseeably and avoidably creates human rights deficits have a duty to reform the institutional order or mitigate the particular deprivations to which they have contributed” [32, p. 33]. Right cosmopolitanism maintains that the global injustice experienced in transnational surrogacy is as a result of social stratification, cultural bifurcation, and unequal international relations in the global world system. The byproduct of such cultural bifurcation is structural inequalities. To close this gap, there is a great need for the elimination of cultural differences and international inequalities; individuals should be conceived of as rational human beings who are entitled to human rights, liberty, autonomy, and human dignity. The ideas of human right, liberty, autonomy, and human dignity are universal moral values that go beyond state borders, with broad implications for global justice and transnational surrogacy.

An implication of the above view for transnational surrogacy is that surrogacy contracts should transcend boundaries, geographical locations, proximity, race, color, and sex. Surrogacy contracts entered into by free and autonomous individuals should be respected and safeguarded by the world community. Since each party has a reproductive right, it does not really matter how such a right is exercised as long as it does not dehumanize and discriminate against people or coerce people into reproductive contracts. It is not the exercise of this right that is the problem but the social structures that such a right operates within. Also, right cosmopolitanism falls short of specifying whose duties it is to act what. To achieve global justice, right cosmopolitanism only broadly suggests overhauling the global system’, which is necessary to realize “one world” that will recognize the human reproductive rights of all, eliminate the North and South divide, and eradicate global inequality.

Unlike cosmopolitanism, communitarianism places weight on the identity and solidarity of individuals within their community. Although communitarians differ in approach, they share the common belief that individuals are part of a “community of interest” [33, p. 107]. Charles Taylor, Alasdair Maclntyre, Micheal Sandel, Michael Walzer, and John Kekes are some of the prominent exponents of this view. In the African interpretation of communitarianism, there are divisions into radical and moderate versions of this view with exponents including John Mbiti, Jomo Kenyatta, Bolaji Idowu, Ifeanyi Menkiti, and Kwame Gyekye [34, p. 107]. Whether in the African or Western sense, the community holds a pivotal place in understanding human nature, rights, and privileges in communitarian constructs. Communitarians believe that “living within … strong qualified horizons [provided by communities] is constitutive of human agency” (quoted in [35, p. 122]). While the individual is understood in relation to the community, the community is placed at the center of value constructs; thus, a bond is formed, “a shared understanding both of the good for man and the good of that community,” in ways that “individuals identify their primary interests with reference to those goods” [36, p. 12].

A consequence of the above for transnational commercial surrogacy is that the right to make decisions about reproduction, what type and with whom, should be restricted within one’s community. Community has the greater obligation to regulate the exercise of such a right in human relationships, since it is the highest moral court of values. In this sense, one could probably say that transnational commercial surrogacy is anti-communitarian and the whole question of achieving global justice within the context of transnational economically motivated surrogacy is bound to end in a cul-de-sac. However, such a supposition does not necessarily lead to any conclusion about the impermissibility of the act of surrogacy within a communitarian ethical framework. Upon further reflection, it is arguable that an Afro-communal ethic would support protecting the interests of surrogate mothers within a community—thereby limiting the exploitation that surrogacy in a transnational order seems to allow.

Turning to neorealism, in a fundamental sense, this perspective diverges from the cosmopolitan and communitarian views with its emphasis on anarchy, self-preservation, and universal domination. Among the more fervent advocates of this view are Kenneth Waltz, Robert Jervis, and John Mearsheimer. Although with some differentiations in their approach, these neorealists seek to overhaul the present international system and its politics. For this reason, they suggest that an international system should operate within an anarchic structure that makes room for a decentralization of power. In lieu of the present hierarchical international system with centralized global governance that exercises authority and provides security and stability, neorealists propose an anarchic international system with a decentralized power structure. Such an international system does not entail disorder, lawlessness, or chaos; it merely implies an absence of centralized global governance [37, p. 88].

The logical inference to draw from the foregoing is that within a neorealist frame of diffusing and decentralizing power in the evolving global order, there would tend to be a contrapuntal narrative against regulating transnational commercial surrogacy. Neorealism presents transnational commercial surrogacy as one of the effects of the present hierarchical international system with centralized world government. However, neorealists would prefer each state to determine and direct its own terms of what constitutes acceptable surrogacy practice in an anarchical structure “where no one commands by virtue of authority” and “no one is obliged to obey” [37, pp. 88–93].

Radical feminists conceive of transnational commercial surrogacy as a form of denigration and objectification of women in a male-dominated world. They hold that transnational commercial surrogacy is morally wrong and should not be permitted, regardless of the desires and motivations of the individuals involved insofar as it is a residue of patriarchy. Within the ideological belief system of radical feminism, “anything developed within the ‘patriarchy’—must be condemned, regardless of the apparent benefits for women” [38, p. 130]. Commercial surrogacy is one such development; hence commercial surrogacy is condemned. While it can hardly ground global justice, or fair and dignified treatment of the surrogate mother and the would-be child, radical feminism instructs that prospective parents should not be allowed through the instruments of the law to purchase the reproductive services of surrogates, since such a reproductive transaction is an alienated labor that denigrates women’s dignity [39]. The problem with surrogacy is not only that it alienates women’s reproductive capacity, but also that it uses the child as a transactional commodity [40]. The child becomes a saleable commodity in a reproductive market. In this way, the “genetic bond” between the surrogate and the child is ruptured [41]; and a parental right which is inalienable becomes alienated.

Following the radical feminist intuition on surrogacy, it might be asked: “Is there sufficient justification for society to deny to adult women the disposition of their reproductive capacities according to their own desires?” [42, p. 229]. Liberal feminism questions the legal prohibition of transnational surrogacy and argues that such a move would violate women’s autonomy and dignity [43]. The force behind liberal feminist theory is self-ownership. Thus, women have rights to their reproductive capability and to pursue happiness. In the liberal feminist thinking, if a surrogacy contract does not have harmful effects on the parties involved or the unborn child, individuals have the freedom to exercise their reproductive rights without interference and beyond the confines of political borders. To the extent that the surrogacy contract is entered into voluntarily, and both parties consent to its terms, the surrogate has an obligation to relinquish the child to the intended parents [44]. One major limitation of this view is that it tends to promote a self-centered theory that considers only the benefits of women in the surrogacy industry with little or no attention to the rights and autonomy of the child.

Ultimately, the views of cosmopolitans, communitarians, liberal feminists, radical feminists, and neorealists on balancing interests, rights, privileges, and resources in the context of cross-border surrogacy are mistaken and not sufficient to address the question of justice undergirding transnational commercial surrogacy in the Global South. Each of these theories drives its view to the extreme. The choice between them becomes a matter of theoretical instability which cannot be arbitrated on any rational ground.

In the next section, we will attempt to develop an Afro-communal theory of social justice as a plausible model for addressing the shortcomings in these theories of global justice. This Afro-communal approach informs Thaddeus Metz’s Afro-communionism, which is a bioethical theory that provides prima facie grounds for the moral worthiness of the practice of commercial surrogacy under a just global system. But before showing how Afro-communal theory fills up this lacuna, it is imperative for us to underscore the principles and ideas of Metz’s interpretation of Afro-communal ethical theory.

## Afro-communalism, transnational surrogacy and the question of global justice

Thaddeus Metz’s Afro-communal theory is rooted in the indigenous views of Africans below the Sahara. The crux of his view is that existing in traditional African societies is “certain kind of relationship” which “is pursued as an end, not merely as a means” [45, p. 117]. A specification for such relationship, he proposes, is based on “identifying with others and exhibiting solidarity” with them [45, p. 118]. The idea of solidarity involves individuals participating in (or being a part of) a community by harmonizing and identifying with other members of the community. Identity deals with individuals’ commitment to improving the quality of life of other members of the community. The notions of identity and solidarity are what Metz referred to as “communion” (or harmony).

Metz builds the idea of communion on relationality, which he sees as producing the moral status of the self, along with other beings and entities. The relational nature of humans is such that “a very large majority of human beings can be subjects of a communal relationship (i.e., they can commune with others), and (nearly) all living human beings can be objects of it, too” [46, p. 167]. The self is realized as a communal being by being in a friendly and loving relationship with other members of the community. In this sense, the “I” becomes part of the “we.” Metz thinks that through compassionate, generous, friendly, and loving attitudes, the self empathizes with other members of the community in what he refers to as “participative empathy,” which aims at self-actualization and human excellence. In his words, “A person exhibits human excellence just insofar as she has character traits that express a prizing of communal or friendly relationships, ones of sharing a way of life with others and caring for their quality of life” [47, p. 105].

Afro-communal justice is built on an idea of relationality that holds that “a being’s moral status is determined by the extent to which it is the kind of thing that can be party to a relationship in which there is communion, understood in terms of identifying with others and exhibiting solidarity with them” [46, p. 166]. In this way, relationality involves both the idea of reciprocity, or an ability of one to be in communion with others who can reciprocate such communion (subjects of communion), and the ability of one to be in communion with those who cannot reciprocate such communion (objects of communion). Metz believes that the ability to be in communion with others is prized much more highly than the ability to merely be “communed with.” For Metz, this ability to be in communion is what determines a communal relationship rooted in solidarity and identity.

Afro-communal justice prizes this communal relationship by promoting human excellence and virtues that create an atmosphere for communion in the community. As relational beings, people have the moral responsibility to exhibit caring, loving, generous, compassionate, empathic, and friendly attitudes toward the well-being of others. In a specific interpretation of African ethics that emphasizes the duties of individuals to the common good of the community, grounded in the relationality of humans and trans-individual nature of morality, Kwame Gyekye articulates:

The success that must accrue to communal or corporative living depends very much on each member of the community demonstrating a high degree of moral responsiveness and sensitivity to the needs and wellbeing of other members. This should manifest itself in each member's pursuit of his duties… The social and ethical values of social well-being, solidarity, interdependence, cooperation, compassion, and reciprocity, which can be said to characterize the communitarian morality, primarily impose on the individual a duty to the community and its members. It is all these considerations that elevate the notion of duties to a priority status in the whole enterprise of communitarian life. [48, p. 118]

For both Gyekye and Metz, the relational status of the individual manifests in some senses of duties and responsibilities. In Gyekye’s ethics of communal responsibility, the good of a person is a function of the performance of duties that promote the communal good. In the same vein, Metz also points out that individuals have the moral responsibility to refrain from attitudes that diminish the moral quality of other persons, which might be injurious to their ability to commune, and to embrace virtuous attitudes—such as helping others in distress, showing concern for others’ needs and welfare, not causing harm to others, and so forth—which promote reciprocal communion, friendship, togetherness, and love in the community. The guiding principle here is that one should behave in such a way that one’s dispositions promote shared identity and communion among people. As Desmond Tutu rightly points out, “social harmony is for us the *summum bonum—*the greatest good. Anything that subverts, that undermines this sought-after good, is to be avoided like the plague. Anger, resentment, lust for revenge, even success through aggressive competitiveness, are corrosive of this good” [49, p. 35].

Following the above conception of moral good in an African communitarian framework, Metz teases out an Afro-communal principle of justice when he writes that “an action is right just insofar as it promotes shared identity among people grounded on good-will; an act is wrong to the extent that it fails to do so and tends to encourage the opposites of division and ill-will” [5, p. 338]. This shows that Afro-communal justice eschews unfriendly actions that reduce the ability of any member of the community to commune. Such actions as “deception, coercion and exploitation” which “fail to honour communal relationships” [50, p. 540] tend to promote the interest of the actor and, in doing so, distance the individual from being in communion with other members of the community. Equity is then realized when individuals take as their article of faith actions that promote solidarity, identity, harmony, and communion.

Furthermore, Metz’s Afro-communal justice acknowledges human dignity and rights. He believes that human dignity and human right are not mutually exclusive. Human dignity deals with the ability of an individual to be in communion with others, while human rights deal with the treatment of respect an individual gives and receives in such a communion. As he writes:

I posit a conception of human dignity according to which individuals have a superlative non-instrumental value insofar as they are in principle capable of entering into community with others. My proposal is that we have human rights insofar as we are beings that can both exhibit friendliness and be treated in a friendly way. [51, p. 311]

In order to respect human dignity, one ought to act in such a way that one’s actions will help others to be in a communal relationship. The maxim here is as follows: Act in such a way that your actions do not degrade others’ “special ability to enter into mutual relationships of identity and solidarity” [51, p. 312]. Unfriendly actions that incapacitate others’ abilities to enter into communal relationships violate their human rights and disrespect their dignity. These human rights violations are unfriendly behaviors that are injurious to communal identity and solidarity, and thus behaviors that individuals ought to refrain from.

However, Metz’s Afro-communal theory has been criticized on various grounds.^6^ While the present paper lacks the space to explore, or even argue against, such criticisms, the immediate task is to articulate how Afro-communalism provides prima facie grounds for the morality of the practice of transnational commercial surrogacy under a just global system. It is necessary for us to state that the current sphere in which transnational commercial surrogacy operates breeds inequality, which veils the true essence of surrogacy. From an Afro-communal standpoint, it undermines the ability of surrogates from the Global South to be in communion with other surrogates from the Global North and those in the Global North to reciprocate such communion.

Transnational commercial surrogacy treats surrogates from the Global South and elsewhere as a means to an end which degrades their ability to enter into friendly relationships with their community, immediate and remote. One way of dealing with such human degradation is to create an atmosphere where the value of communion is highly priced. In such an atmosphere, the severe dissonance experienced by surrogates in the Global South, as well as those from the Global North, could be addressed by “treating people as special in virtue of their capacity to commune” [45, p. 125]. This approach, in a way, makes good sense of why there is a strong moral reason for global commercial surrogacy to be based on friendliness, love, and communion, beyond consideration of monetary incentives [56]. A starting point for creating such an atmosphere is to construe surrogates as individuals with the ability to enter into communal relationships with others, and “whenever one encounters an individual with the requisite degree of the capacity for sharing a way of life and caring for others’ quality of life, one must treat that capacity of hers with equal respect” [57, pp. 544–545]. To the extent that surrogates have relational capacities, they are to be treated with respect because of their ability to identify and be in solidarity with others.

This perspective entails that intended parents in the Global North should act in friendly, loving, and reciprocal ways, whereby their actions would help surrogates in the Global South to commune with them. In the same vein, surrogates in the Global North, such as a Latina hired in Los Angeles, are enjoined to base the contractual agreement more on the primacy of virtues of reciprocal friendliness and communion. Such reciprocal engagement would not prize contractual agreement for its own sake as a claim to outright entitlement to the child once compensation has been sealed. In an Afro-communal ethical framework, compassion, sympathy, empathy, solidarity, and friendliness would synergistically constitute the language and the defining parameters of engagement in surrogacy contracts between would-be parents and would-be transnational surrogates.

In line with this, it is possible that surrogacy entails a shared responsibility that would promote harmony and togetherness within a transnational context. This shared responsibility involves, first, the responsibility of the surrogates to their unborn child. Surrogates have the biological responsibility to look after the fetus while in the womb by living a healthy lifestyle. Second, it involves the responsibility of the intended parents to the child. Regardless of whether they have genetic link to the child or not, intended parents have the responsibility to commune with the child, even though the child cannot reciprocate such communion. Finally, it involves the responsibility of the intended parents to the surrogates and vice versa. This implies that intended parents and surrogates are not merely contractual parties but relational entities that are in communion with one another. Metz’s concept of harmony, which is “achieved through close and sympathetic social relations within the group” [58, p. 3], holds that both intended parents and surrogates are in a harmonious relationship.

Essentially, when couples that wish for a child employ a surrogacy contract to shy away from meeting the needs of surrogates, caring only about their prospective child and breaking away from the surrogates once the child is received, they undermine their communication and relationship with the surrogates, which extends even after birth,; in such cases, relinquishment of the child should be frowned upon. In order to promote communion, intended parents need to be sensitive to the needs of the surrogates not only during but also after birth, such as through provision of post-partum care to surrogates, continued contact, and so on. Surrogates should, in turn, reciprocate by exhibiting an attitude of openness, truthfulness, friendliness, and love. This would limit the present exploitation in the global order, since the interests of the surrogates, child, and intended parents would be protected within the global community.

Not only do surrogates have the capacity for friendliness, but they also help others to be in communion. Surrogates have a “sympathetic understanding” of the pain of infertile couples and the dangers infertility poses to their ability to be in friendly communion with others. As an attempt to foster communion, surrogates’ decision to carry the child of infertile couples as a means of producing as much friendliness as possible is somewhat morally justifiable. Accordingly, the action of surrogates is justifiable insofar as it enhances the abilities of others to enter into “mutual relationships of identity and solidarity” and unjustifiable insofar as “it degrades a person’s special ability to enter into mutual relationships of identity and solidarity” [51, pp. 311–312].

One of the fears often expressed about transnational surrogacy is that it ruptures the genetic link between the surrogate and her child. While genetic links might be necessary, they are not sufficient representations of the totality of parenthood. Apart from these genetic links, the intentions of the intended parents to rear, nurture, care for, and love their prospective child are also relevant to parenthood. Parenthood is about not only natural and biological links but also social links [59]. As Metz rightly captures:

Social ties matter morally, while biological ties do not. More specifically, according to the communal principle, normal adults have a dignity in virtue of their capacity to be party to relationships of identity and solidarity. Treating them with respect means (in part) enabling them to actualize this capacity and taking care not to interfere with their actualizations of it, i.e., with the relationships themselves. And there is nothing genetically essential to these relationships, ones of enjoying a sense of togetherness with others, participating with them on a cooperative basis, and helping them out of sympathy and for their sake. [60, p. 60]

Pursuant to the above, one can infer that it is morally permissible for surrogates to give birth to children who are to be cared for by the commissioning couples as long as doing so prevents social disharmony.

Good institutions that advance communion among individuals are essential to the promotion of best practices in commercial surrogacy. These will create a good structure whereby individuals identify with and exhibit solidarity, friendliness, love, and communion toward others. In addition to the Afro-communal normative prescription for a just global order in transnational commercial surrogacy, it is important that there is firm and enforceable national and regional legislation on local and international commercial surrogacy. This is important in order to minimize the exploitation of vulnerable impoverished women in the Global South and elsewhere. Solidarity is a core value that can improve the quality of life of surrogates from the Global South and elsewhere. If guided by the Afro-communal ethical principle of solidarity, surrogates in the Global South are likely to enjoy a sense of togetherness and identity with surrogates in the Global North when their quality of life is improved. With structures of identification and solidarity, reinforced by new digital social media, individuals can enjoy a sense of togetherness while engaging in global commercial surrogacy.

## Conclusion

In this paper, we engaged the views of cosmopolitans, communitarians, liberal feminists, radical feminists, and neorealists on the question of justice in transnational commercial surrogacy. These perspectives are essentially problematic for realizing social justice in the transnational surrogacy phenomenon. As an alternative, we proposed Metz’s interpretation of the Afro-communal notion of justice in order to ground the moral permissibility of transnational surrogacy without attenuating the question of global justice. In the light of an Afro-communal ethics, it is argued that transnational surrogacy is morally permissible under a just global order built on the values of communion. Such a global system creates a friendly atmosphere where surrogates can identify with others and foster solidarity. This perspective deserves to be taken seriously in contemporary discourses on transnational surrogacy and global justice.

## Footnotes

1 Our usage of the terms Global North and Global South in this paper is not to imply that the gap between them is monolithic. While globalization has in many ways served to reinforce the divide between the Global North and South, it has also worked to temper it. Just as globalization has brought about the deterioration of middle-class and working-class living conditions in the Global North, so has it enabled the establishment of a larger middle class and expanding upper class in India and China and, to a certain degree, in Brazil and South Africa. So even though the divide between the Global North and South is not flat and there are some overlaps, this paper underscores their cleavage in the context of surrogacy to show that demand for surrogacy is dominant in the Global North while the Global South serves as the dominant supply chain.

2 For cosmopolitans, the elimination of such structural inequalities and redistribution of wealth will guarantee global justice. Contrarily, communitarians and neorealists both insist—albeit differently—that state-centric approaches to transnational commercial surrogacy must be adopted in order to achieve global justice. Liberal feminists endorse surrogacy not only as an expression of sexual rights and freedom from patriarchal structures but also as a veritable conduit of women’s empowerment. Radical feminists see surrogacy as another form of the patriarchal alienation of women from their true essence.

3 The famous *Baby M* case was the custody trial in US between surrogate Mary Beth Whitehead and intended parents, William and Elizabeth Stern, in the 1980s. Days after the birth of *Baby M,* Mary Beth Whitehead refused to give up her parental right to the baby because she could not overcome the love and bond she shared with *Baby M*. Out of desperation for parenthood after numerous attempt to persuade Mary Beth Whitehead to relinquish her parental right to *Baby M* and let William and Elizabeth Stern keep her, Sterns sued to have the agreement between them and Mary Beth Whitehead enforced.

4 Research by Oxfam [12, 17] has shown that the richest eighty-five people in the world own as much as the poorest half of the global population. Between 2013 and 2014, these 85 people grew $668 million richer every day. The report observes, “If Bill Gates were to cash in all his wealth, and spend $1 m every single day, it would take him 218 years to spend it all. In reality though, he would never run out of money: even a modest return of just under two percent would make him $4.2 million each day in interest alone” [17, p. 8].

5 In his paper, Krasner examines different roles played by actors and their consequences in the international economic system. He claims that the international economic regime is characterized by a “hegemonic maker of the system” as well as breakers and takers of the system. He argues that the makers of the system, like the United States, through power and policies, shape the international economic regime. Medium-size states are the breakers of the system: although they lack power to shape the international economic regime, they could undermine the order established by this regime. The small states are takers who “can adjust their own policies to try to maximize their particular objectives, but, for them, the general nature of the regime is a given” [18, p. 636].

6 See, for instance, [52–55]. In addition to such criticisms, this paper is of the view that Metz has not shown how his theory of dignity based on capacity is descriptively identifiable with any African society. The idea appears more as a normative construct to fill a lacuna created by the failure of the descriptive theories of individual rights in Africa to account for the source of such rights, which could be dignity or something else. To that extent, his Afro-communal view of dignity appears more Metzian than African.
